# Oblique distribution patterns and the underlying mechanical model of orebody groups controlled by structures at different scales

**DOI:** 10.1038/s41598-024-55473-z

**Published:** 2024-02-26

**Authors:** Runsheng Han, Jianbiao Wu, Yan Zhang, Qing Chen, Bangtao Sun

**Affiliations:** 1 Southwest Institute of Geological Survey, Geological Survey Center for Nonferrous Metals Resources, Kunming University of Science and Technology, Kunming, 650093 Yunnan China; 2 Yunnan Chihong Zinc Germanium Co., Ltd, Qujing, 655011 Yunnan China; 3 Yiliang Chihong Co., Ltd, Zhaotong, 657600 Yunnan China

**Keywords:** Economic geology, Structural geology

## Abstract

The oblique distribution of orebodies is a basic feature of the spatial distribution of orebody groups in hydrothermal deposits, and it is closely related to the shearing effect. However, the oblique distribution patterns of orebody groups controlled by ore-controlling/ore-forming structures at different scales (orebody, ore deposit and ore field scales) and the underlying formation mechanism are unclear but could be used to directly constrain mineral exploration and prospecting breakthroughs in the deep and peripheral areas of ore deposits. This paper uses the northeastern Yunnan Ge-rich Pb–Zn ore concentration area in the Sichuan–Yunnan–Guizhou metallogenic area as an example to analyse and demonstrate the oblique distribution patterns of orebodies (orebody groups) controlled by ore-forming/ore-controlling structures at different scales and the underlying mechanical model based on the Theory and Methods of Ore field Geomechanics. The results indicate that in 3D space, the oblique distributions of orebodies (veins), orebody groups and ore deposits are controlled by the mechanical properties, kinematic characteristics, and tectonic stress fields of ore-forming/ore-controlling fault structures of different sequences during the mineralization period. This study has an important guiding role for ore field-scale exploration deployment, evaluation of deep and peripheral prospecting, and exploration project arrangement, with the aim of helping mining companies increase resource reserves and production.

## Introduction

In nonmagmatic or magmatic hydrothermal deposits, the oblique distribution of orebodies is a basic feature of the spatial distribution of orebody groups and has a genetic connection with shearing. In hydrothermal deposits, the gaps in various structures, such as primary structures of intrusions, sedimentary structures, post-deformation structures, and contact zone structures, are not only channels for the migration of ore-bearing hydrothermal fluids but also places for mineral precipitation. During the mineralization period, the relative movement of these structures under stress not only provides impetus for the migration of ore-bearing hydrothermal fluids and the formation of ore-bearing spaces but also provides the physical and chemical conditions for mineral precipitation. However, at present, the oblique distribution patterns of orebody groups and the formation mechanism of oblique distributions are unclear. In addition, models of such oblique distribution patterns and the underlying mechanical model of orebody groups controlled by structures at different scales have rarely been studied, but these patterns could be used to directly constrain mineral exploration and prospecting breakthroughs in deep and peripheral areas of ore deposits. Therefore, exploring the oblique distribution patterns and underlying formation mechanisms of orebodies has an undoubtedly important guiding role in ore prospecting prediction and exploration project arrangement in deep and peripheral areas of ore deposits.

**Figure 1:**
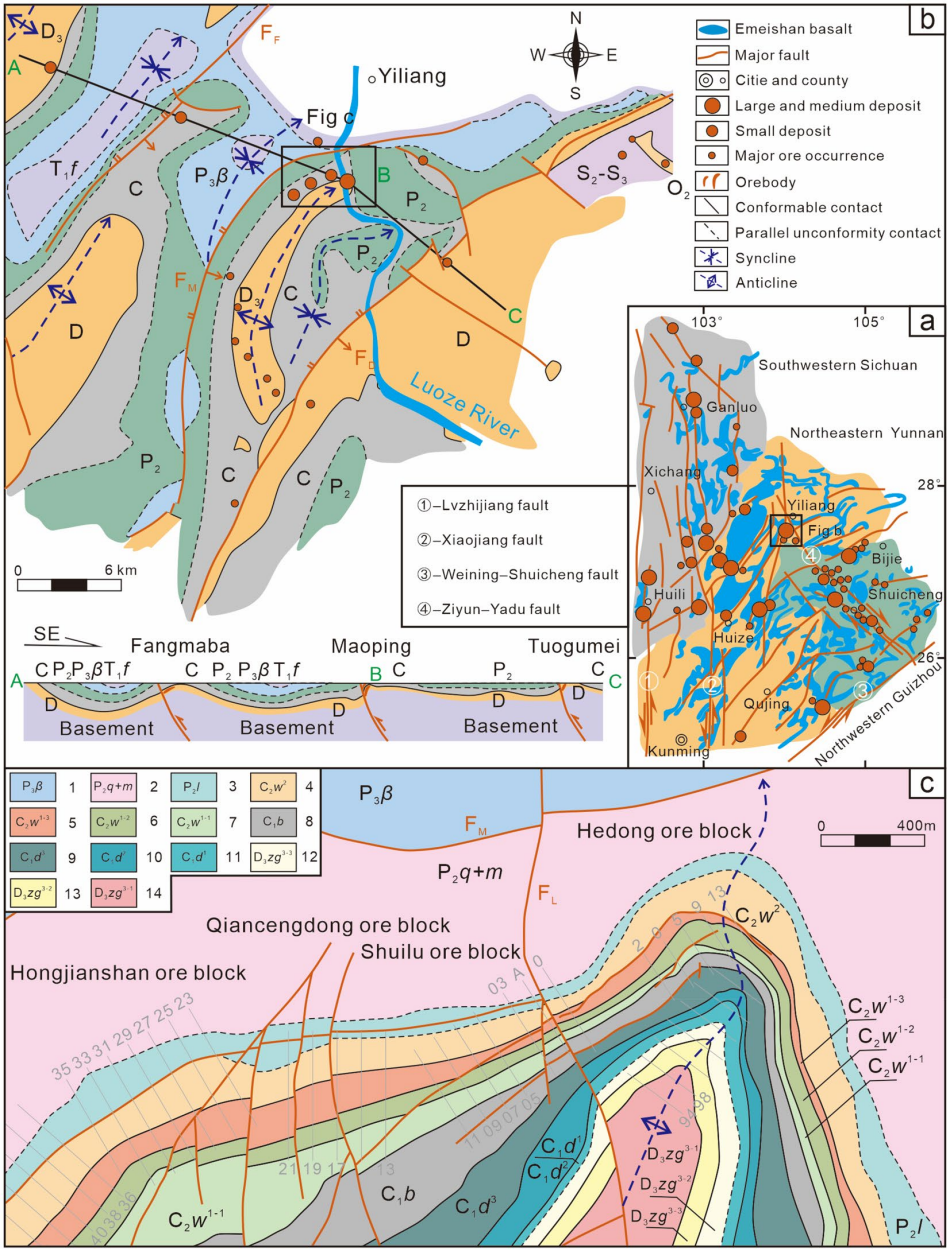
Distribution map of the main ore-controlling structural belts and large- and medium-sized ore deposits in the Sichuan–Yunnan–Guizhou Pb‒Zn polymetallic metallogenic area (**a**), Geological map of Maoping Pb–Zn ore field (**b**) and deposit (**c**) (^1^ redrawn according to^2^). 1–Middle Triassic Guanling Formation, 2–Lower Triassic Yongningzhen Formation, 3–Lower Triassic Feixianguan Formation, 4–Upper Permian Xuanwei Formation, 5–Upper Permian basalt formation, 6–Middle Permian Qixia + Maokou Formation, 7–Lower Permian Liangshan Formation, 8–Middle Carboniferous Weining Formation, 9–Third submember of the first member of the middle Carboniferous Weining Formation, 10–Second submember of the first member of the middle Carboniferous Weining Formation, 11–First submember of the first member of the middle Carboniferous Weining Formation, 12–Lower Carboniferous Baizuo Formation, 13–Third member of the lower Carboniferous Datang Formation, 14–Second member of the lower Carboniferous Datang Formation, 15–First member of the lower Carboniferous Datang Formation, 16–Third submember of the third member of the Upper Devonian Zaige Formation, 17–Second submember of the third member of the upper Devonian Zaige Formation, 18–First submember of the third member of the Upper Devonian Zaige Formation. Distribution map of the main ore-controlling structural belts and large- and medium-sized ore deposits in the Sichuan–Yunnan–Guizhou Pb‒Zn polymetallic metallogenic area (**a**), Geological map of Maoping Pb–Zn ore field (**b**) and deposit (**c**) © 2024 by Runsheng Han is licensed under Attribution 4.0 International.

The Ge–Pb–Zn ore concentration area in northeastern Yunnan is an important part of the Sichuan–Yunnan–Guizhou Pb–Zn polymetallic metallogenic area (Fig. 1a,^1^ redrawn according to^2^). There are more than 350 Ge-rich silver-Pb–Zn polymetallic deposits and ore (mineralization) occurrences represented by the Huize and Maoping superlarge deposits. The ore deposits are obviously controlled by fault–fold structures and favourable lithological associations, with “rich (ultrahigh grade), large (large-scale ore deposits and orebodies), many (many associated elements such as Ge, Ag, and Cd, and many ore-hosting strata), deep (large depth extension), strong (strong hydrothermal alteration), high (high metallogenic temperature), and clear (clear zoning of mineral and alteration associations)” characteristics^3^. Thus, this area has long been a focus of attention and research in the geoscience community^1,3–8^. Studies have been carried out in the areas of mineralization chronology^2,3,9–15^, the origin of ore deposits^2,3,7,15–19^, tectonic ore-controlling patterns^20–25^, and prospecting prediction^1,3,6,7,26,27^; important research progress and major breakthroughs in deep prospecting have been made in metallogenic theory, R&D and exploration technology application.

The Sichuan–Yunnan–Guizhou Pb–Zn polymetallic metallogenic area is located on the southwest margin of the Yangtze Block at the junction of the Tethys metallogenic domain and the Pacific Rim metallogenic domain (Fig. 1a,^1^ redrawn according to^2^)^7,28–32^. This area is bounded to the west by the N–S-trending Anninghe‒Lvzhijiang Fault, to the north by the NW-trending Kangding‒Yiliang‒Shuicheng Fault, and to the south by the NE-trending Mile‒Shizong‒Shuicheng Fault (Fig. 1a,^1^ redrawn according to^2^). Regionally, these three faults are closely related to Pb–Zn deposits. The formations in the area are well developed and consist of metamorphic basement and sedimentary cover^7,33^. The area is characterized by frequent and long-lasting magmatic activity that occurred from the Jinning to Yanshan periods^10,28,34,35^. Magmatic rocks are widely but unevenly distributed. Among them, the Haixi period Emeishan basalt not only has a wide distribution area but also a large thickness, forming the famous Emeishan volcanic province^10^. There are 15 main fault structures in the area, divided into three groups: (nearly) N–S-trending, NW-trending, and NE-trending. The (nearly) N–S-trending faults are distributed mainly in the southern Sichuan and northern Yunnan regions between the Xiaojiang Fault and the Anninghe‒Lvjujiang Fault^7^. The NW-trending faults are distributed in the northwestern region of Guizhou Province between the southwestern Kangding‒Yiliang‒Shuicheng Fault and northeastern Yunnan. The NE-trending faults are mainly distributed in the northeast region of Yunnan Province to the northwest of the Mile‒Shizong‒Shuicheng Fault. A series of NE-trending structural belts are distributed from south to north, forming a "xi-type" structure accompanied by NW-trending extensional (torsional) faults.

Therefore, in the present study, the Maoping superlarge Ge-rich Pb‒Zn deposits in the Ge-rich Pb‒Zn ore concentration area in northeastern Yunnan Province are taken as a typical example to reveal the oblique distribution patterns of orebody groups and the underlying mechanical model based on the Theory and Methods of Orefield Geomechanics^36^.

## Geological setting

The Maoping Ge-rich Pb–Zn ore field is located at the intersection of the NE-trending Huize–Niujie oblique thrust–strike-slip fault–fold belt, the N–S-trending Qujing–Zhaotong concealed fault zone, and the NW-trending Ziyun–Yadu deep fault zone (Fig. 1b). A series of NE-trending compound overturned anticline groups are derived from the hanging walls of three parallel distributed faults, i.e., the Fangmaba Fault, Maoping Fault, and Daibu Fault. These faults and anticlines jointly controlled the ore field^1,25^.

The Maoping deposit is controlled mainly by the NE-trending Maoping sinistral compressional–torsional fault and the Maomaoshan compound overturned anticline in its hanging wall (Fig. 1c). The main structures in the deposit are NE-trending, NW-trending and N–S-trending structures, with the NE-trending interlayer sinistral compressional–torsional fault dominating. The deposit is mainly distributed within the Devonian, Carboniferous, and Permian carbonate–clastic rock series, which are mostly in parallel unconformable or conformable contact. The magmatic rocks are mainly in the upper Permian Basalt Formation. The orebodies mainly occur in the greyish-white to dark grey fine–medium crystalline dolomite of the upper Devonian Zaige Formation (D_3_*zg*), the light flesh-red or greyish-white massive fine crystalline dolomitic limestone of the lower Carboniferous Baizuo Formation (C_1_*b*), and the light grey to dark grey medium- to thick-layered dolomitic limestone of the upper Carboniferous Weining Formation (C_2_*w*)^25^.

The deposit is composed of the Nos. I, II, III and VI orebodies east of the Luoze River (known as the Hedong ore block), and the Shuilu, the Qiancengdong and the Hongjianshan ore blocks west of the Luoze River (known as the Hexi ore block). The No. I orebody group is located within the interlayer fault of the D_3_*zg*^*3–2*^ Formation, and it trends NE‒SW, dips to the SE, has a dip angle of 60°–85°, and a pitch to the SW. The No. II and No. III orebody groups are located within the interlayer fault of the C_1_*b* and C_2_*w*^*1–2*^ Formations, and they trend NE‒SW, dip to the SE, and have a dip angle of 60°–90° and a SW pitch. The No. VI orebody group is located within the interlayer fault of the D_3_*zg*^*3–1*^ Formation, and it trends NE‒SW, dips to the SE, and has a dip angle of 50°–70° and a SW pitch. The orebodies in the Shuilu, Qiancengdong, and Hongjianshan ore blocks are located within the interlayer fault zone of the C_2_*w*^*1–2*^ Formation, and they trend NE‒SW, dip to the NW, and have dip angles of 70°–85° and a NE pitch^25^.

The average Pb + Zn grade of the orebody is greater than 25%; for example, at present, the I-6 orebody is the largest orebody in the deposit, with Pb grades between 4.82 and 8.71% and an average grade of 7.02%. The average Zn grade ranges from 14.62 to 19.81%, with an average grade of 16.93%. The average Ge content ranges from 10.06 to 39.22 ppm. The orebodies are mainly lenticular, vein-like and stratiform-like. The main ore minerals are galena, sphalerite and pyrite, and the gangue minerals are dolomite, calcite, a small amount of quartz and barite. The ore structures are compact massive, disseminated, vein-like, veinlet-like, massive and stellate, and the ore textures are mainly granular and metasomatic. The main types of wallrock alteration include pyritization, ferritization, dolomitization, calcification and silicification^25^.

## Materials and methods

Based on regional tectonic evolution, we revealed the structural hierarchal ore-controlling patterns of the Sichuan–Yunnan–Guizhou Pb‒Zn polymetallic metallogenic area and determined the ore-forming structure association styles at different scales.

The Theory and Method of Orefield Geomechanics^36^ were applied to analyse the geometric, kinematic, mechanical, and material characteristics of structures at different scales within the deposit, and the spatial distribution characteristics of known orebodies or mineralized bodies were combined to identify the mineralized structures.

Based on the principle of structural sequence transformation^36^, the mechanical properties of ore-forming structures and their secondary structural planes are analysed using a stress unit body, revealing the control effect of principal compressive stress on the formation of ore-bearing spaces and summarizing the controls on the spatial distributions of orebodies, orebody groups, deposits, and ore fields.

Based on the mechanical properties and oblique distribution patterns, the distributions of orebody groups at the deposit scale and the distributions of orebodies at the orebody groups are predicted.

### Structural hierarchal ore-controlling patterns and ore-forming structure association styles

#### Structural hierarchal ore-controlling pattern

Han et al. suggested that the main ore-controlling factors in the Sichuan–Yunnan–Guizhou Pb‒Zn polymetallic metallogenic area are fault–fold structures and favourable lithological associations, with fault–fold structures being the typical ore-forming structural planes of this type of deposit^1,3^. In addition, the Ge-rich Pb‒Zn deposits are the product of the infiltration of ore-forming fluids driven by fault–fold structures to form favourable ore-forming structures along favourable rock associations, creating a zonal intracontinental tectonic system with high diversity in the area (Fig. 1).

**First-order (the Sichuan–Yunnan–Guizhou Pb‒Zn polymetallic metallogenic area):** A NE-trending sinistral oblique thrust strike-slip fault–fold belt formed in northeastern Yunnan ore concentration area, a NW-trending syncline strike-slip fault–fold belt formed in the northwestern Guizhou ore concentration area, and a nearly N‒S trending sinistral strike-slip fault zone and its derivative WNW–ESE to E–W-striking strike-slip fault–fold zone formed in the southwestern Sichuan ore concentration area.

**Second-order (Northeastern Yunnan ore concentration area):** The Pb‒Zn polymetallic ore concentration area controlled by eight NE-trending fault–fold structural belts is distributed in a sinistral oblique manner.

**Third-order (ore field):** Several faults within the controlled ore concentration area jointly controlled each ore field, such as the Maoping Pb–Zn ore field controlled by the Fangmaba Fault, Maoping Fault and Daibu Fault.

**Fourth-order (deposit):** A NE-trending fault–fold structure association controls the right echelon distribution of a series of Pb–Zn deposits.

**Fifth-order (orebody group):** An interlayer fault zone in multiple ore-hosting strata in the hanging wall anticline of the main controlling fault of the fault–fold structure causes the orebody groups to present a sinistral oblique distribution.

**Sixth-order (orebody):** An ore-bearing fault zone in a single ore-hosting stratum causes the ore veins (orebodies) to be distributed in a sinistral oblique manner.

#### Ore-controlling/ore-forming structure association styles at different scales

**Figure 2:**
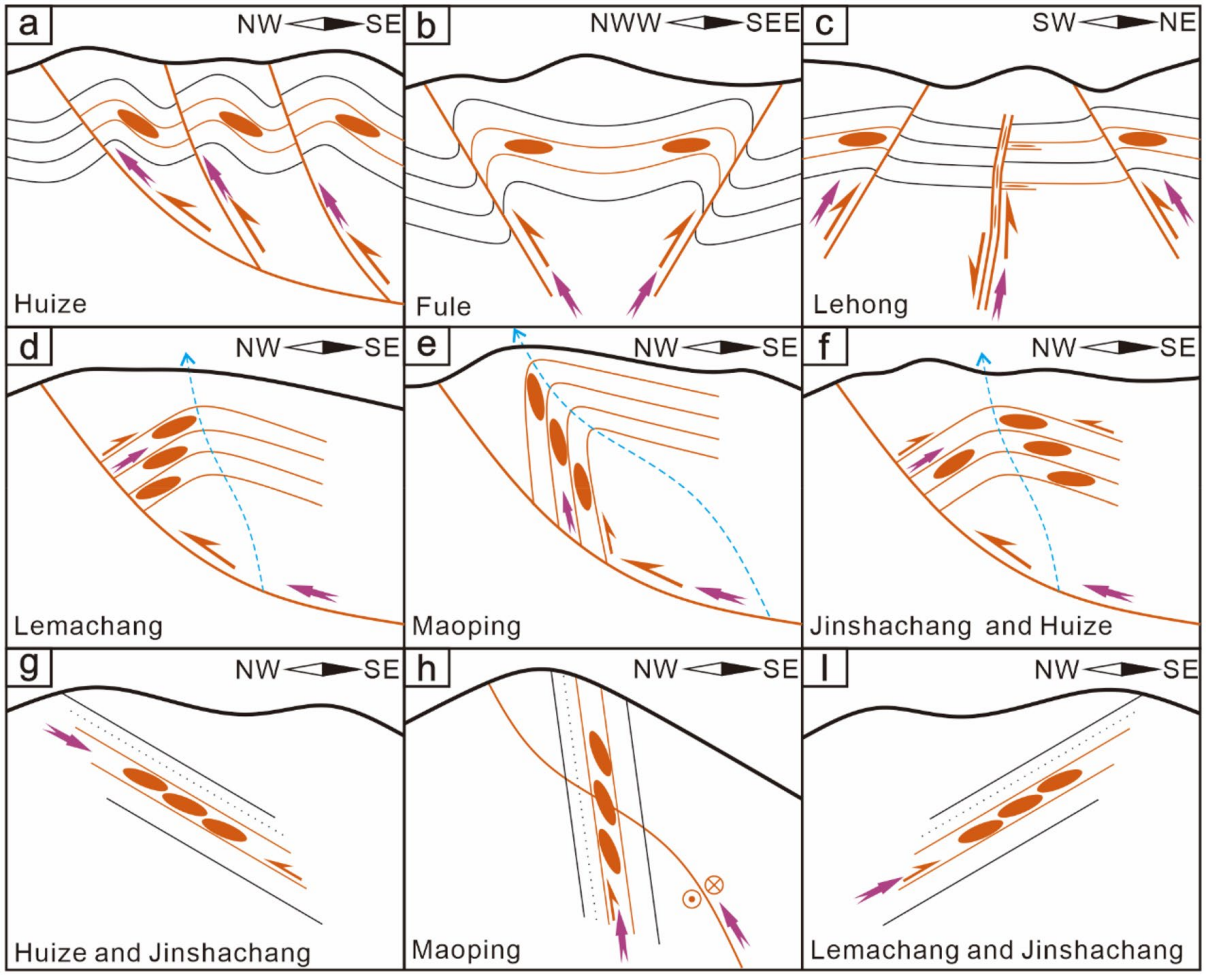
Ore field scale (**a**–**c**), deposit scale (**d**–**f**) and orebody group scale (**g**–**i**) ore-control/ore-forming structure association styles.

In the Sichuan–Yunnan–Guizhou Pb‒Zn polymetallic metallogenic area, the ore-controlling/ore-forming structures of different scales have different association styles (Fig. 2). In summary, for the Pb–Zn ore fields in northeastern Yunnan ore concentration area, the main ore-controlling structure is an oblique thrust strike-slip fault–fold zone, and the ore-controlling structure association styles include mainly homocline-type oblique thrust strike-slip fault–fold structure associations (Pb–Zn deposits in Huize, Maoping, etc.), diagonal-type oblique thrust strike-slip fault–fold structure associations (Pb–Zn deposits in Maozu, Fulechang, etc.), and anticline-type oblique strike-slip fault–fold structure associations (Pb–Zn deposits in Lehong, etc.). At the deposit scale, the main ore-forming structures include fault–fold structure associations, and the style of the ore-forming structure association is the thrust fault–anticline association (Pb–Zn deposits in Lemachang, Jinshachang, etc.). At the orebody scale, the main ore-bearing structure style is the oblique secondary ore-bearing fault‒fissure zone (Pb–Zn deposits in Maoping, Huize, etc.).

Therefore, whether a tectonic hierarchical ore-controlling pattern or an ore-controlling/ore-forming structure association style is used, ore-controlling/ore-forming structures at different scales have sequential genetic connections.

### Spatial distribution patterns and the underlying mechanical model of deep orebody groups

**Figure 3:**
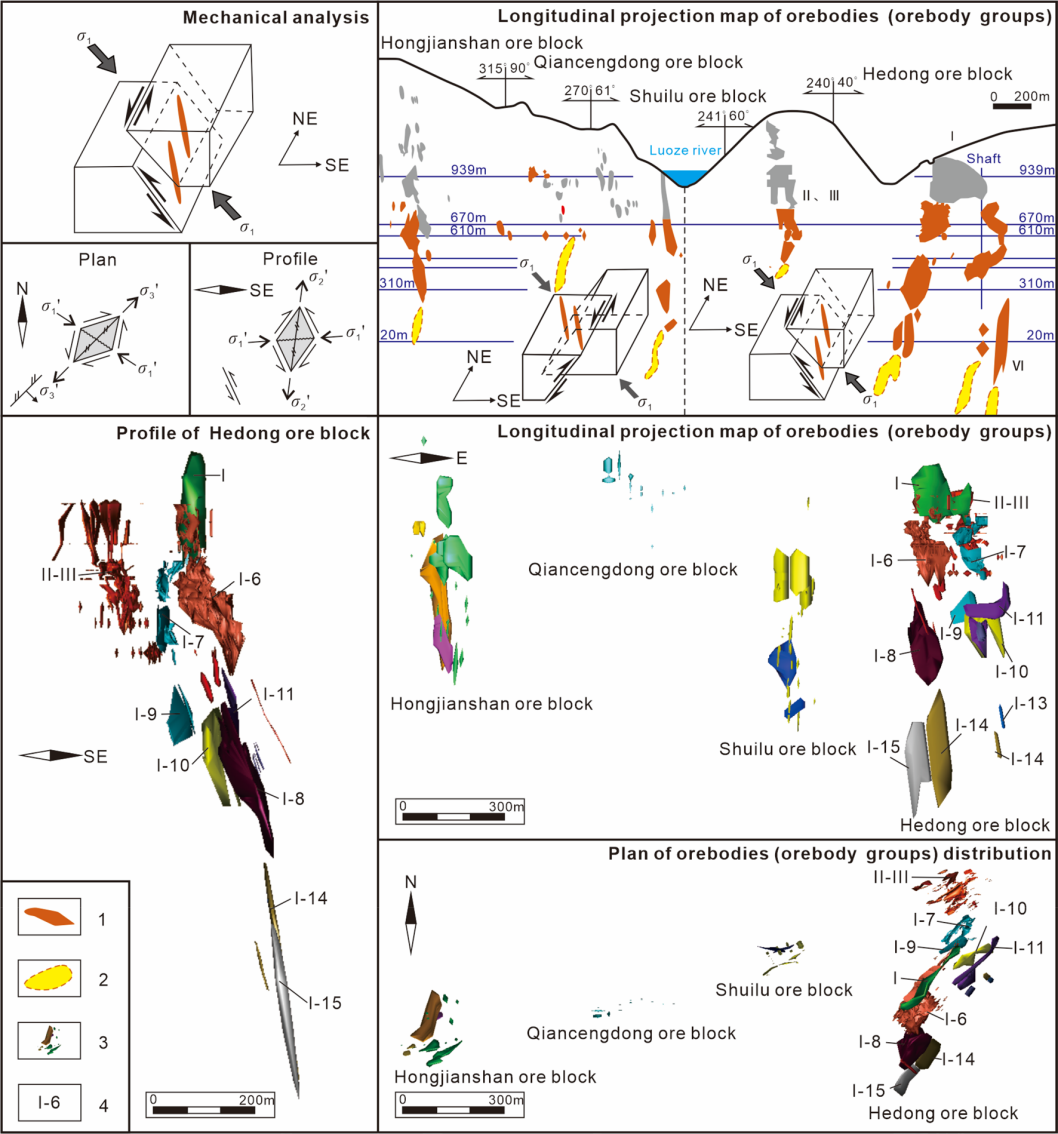
The underlying mechanical model of the spatial distribution of the deep orebodies and longitudinal projections of the Maoping Pb–Zn deposit. 1–known orebodies, 2–predicted orebodies, 3–3D display of known orebodies. 4–Known orebodies number. The underlying mechanical model of the spatial distribution of the deep orebodies and longitudinal projections of the Maoping Pb–Zn deposit. © 2024 by Runsheng Han is licensed under Attribution 4.0 International.

Studies show that the spatial distribution of orebodies in hydrothermal deposits is controlled by the mechanical and kinematic mechanisms of ore-bearing faults during the mineralization period. In northeastern Yunnan ore concentration area, the main ore-bearing faults that control the distribution of the main orebodies are the interlayer compressional-torsional and torsion-compressional faults of the hanging wall anticlines of the NE-trending syncline thrust strike-slip fault–fold zone, making the downdip length an orebody group greater than its along-strike length. In addition, the mechanical properties and kinematics of the ore-bearing faults directly control the distribution characteristics of the main orebody in the pitch direction (Fig. 3), and the pitch direction is consistent with the movement direction of the downthrow of the ore-bearing faults. Therefore, the main orebody group of each deposit in this ore concentration area is distributed in the sinistral compressional-torsional interlayer slip zone of the hanging wall of the main fault in the NE-trending fault–fold zone, and the main orebody group pitches SW. For example, consider the Maoping Pb‒Zn deposit.

**Figure 4:**
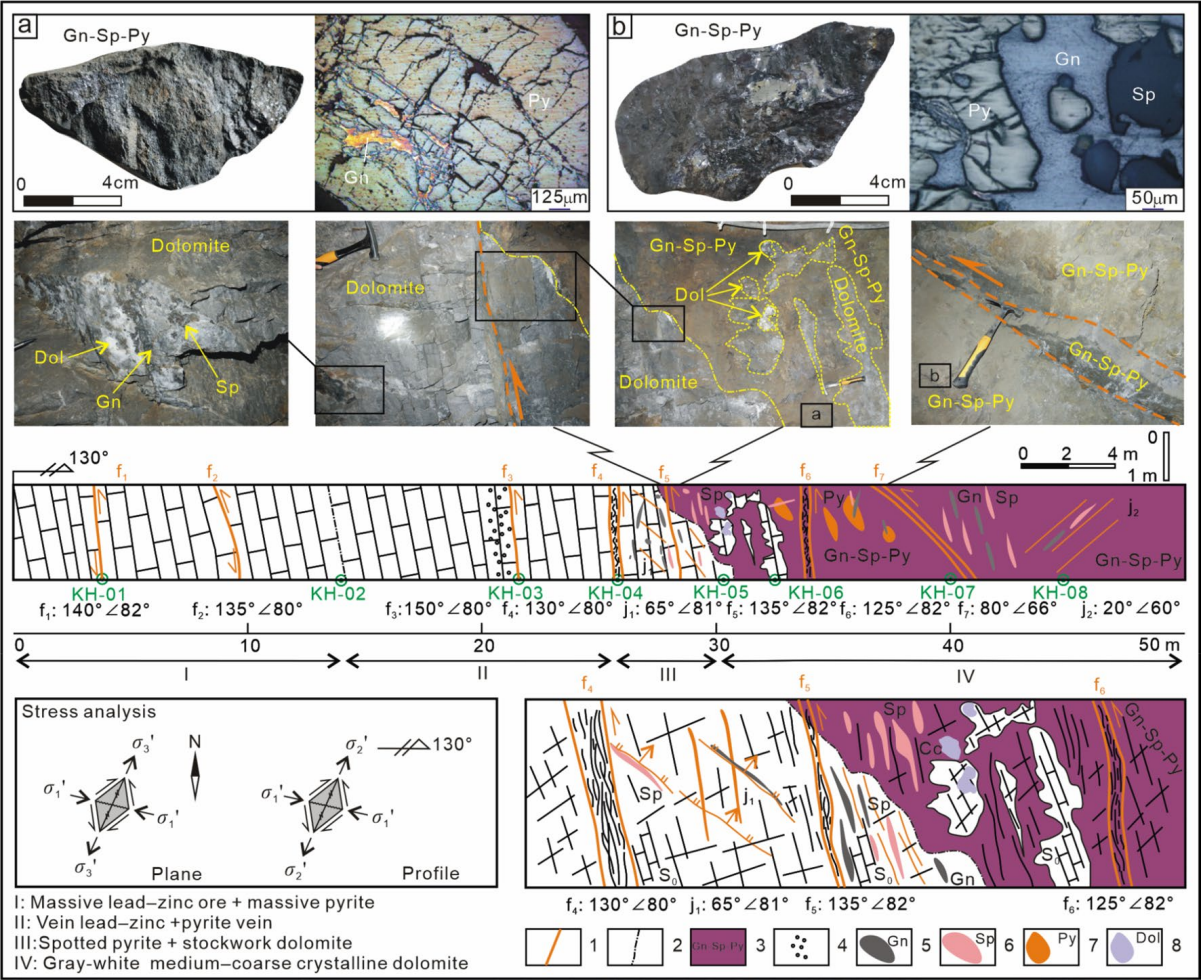
Metallogenic structures and orebody characteristics^25^. Geological profile of the I-6 orebody of 683 m along the line 98 + 1 in the Hedong ore block of the Maoping Pb–Zn deposit.1–Fault, 2–Alteration boundary, 3–Orebody. 4–Dissolution pore, 5–Galena (Gn), 6–Sphalerite (Sp), 7–Pyrite (Py). 8–Dolomite (Dol).

**Figure 5:**
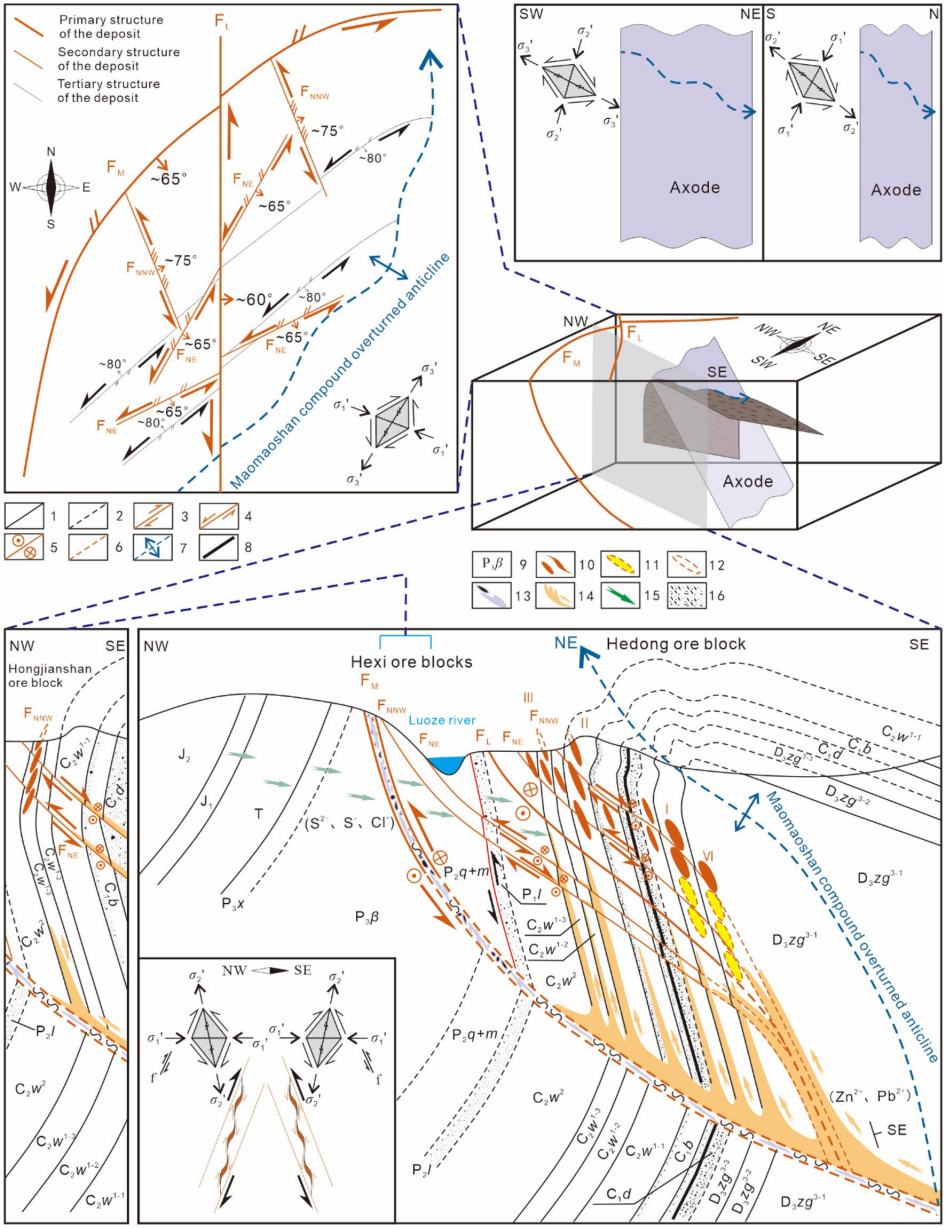
Comprehensive pattern map of ore-controlling of structure of the Maoping Pb–Zn deposit^25^. 1–Integrated contact, 2–Parallel unconformity contact, 3–Compressive fault, 4–Tensile fault, 5–Torsional fault, 6–Inferred fault, 7–Anticline, 8–Coal-bearing, 9–Formation code, 10–Known orebodies, 11–Predicting orebody, 12–Denuded orebody, 13–Dolomitization and its cemented limestone breccia, 14–Ore-forming metals fluids, 15–Reducing fluids, 16–Argillaceous and sandy clastic rock.

The spatial distribution patterns of the deep orebody groups and the underlying mechanical model are analysed based on the structural hierarchal ore-controlling patterns and ore-forming structure association styles. By applying the theory and method of ore field geomechanics^36^, through a fine analysis of the ore-controlling structures (Fig. 4) (additional research can be found in^25^), the structural framework of the Maoping Pb–Zn deposit was determined. The deposit is controlled by the oblique thrust-strike-slip fault and its derived compound anticline structural belt of dolomitized bodies of thermally brecciated rocks. The main controlling factors of the deposit are structural and combinations of lithological factors. The ore-controlling structures of the deposit are classified into four grades. The NE-trending Maoping sinistral compressional-torsional fault and the N–S-trending Luozehe sinistral torsional fault are fourth-order structures. The NE-trending Maomaoshan compound overturned anticline, the NNW-trending sinistral transtensional fault and the NE-trending in-sequence compressional-torsional fault derived from fourth-order structures are fifth-order structures. The NE-trending interlayer sinistral compressional-torsional faults are sixth-order structures. On this basis, we summarize the ore-controlling rules of these structures: the NE-trending Maoping sinistral compressional-torsional fault and the NE-trending Maomaoshan compound overturned anticline together form an oblique thrust–strike-slip fault–fold belt, which directly controls the spatial distribution of the deposit. The analysis results reveal that (1) the ore-conducting structure of the deposit is the NE-trending Maoping sinistral compressional-torsional fault; (2) the ore-distributing structures are the N–S-trending Luozehe sinistral torsional fault, the NE-trending Maomaoshan compound overturned anticline, the NNW-trending sinistral torsional fault and the NE-trending in-sequence compressional-torsional fault; and (3) the ore-hosting structures are the NE-trending interlayer sinistral compressional-torsional fault and the lower-order joints and fissures. On the basis of these results, we further constructed an oblique thrust–strike-slip fault–fold structure ore-controlling model (Fig. 5)^25^, providing a basis for analysing the oblique distribution patterns and the underlying mechanical model at different scales.

### Vein groups in the ore-bearing fault at the orebody groups scale

#### Planar distribution patterns of the vein groups

**Figure 6:**
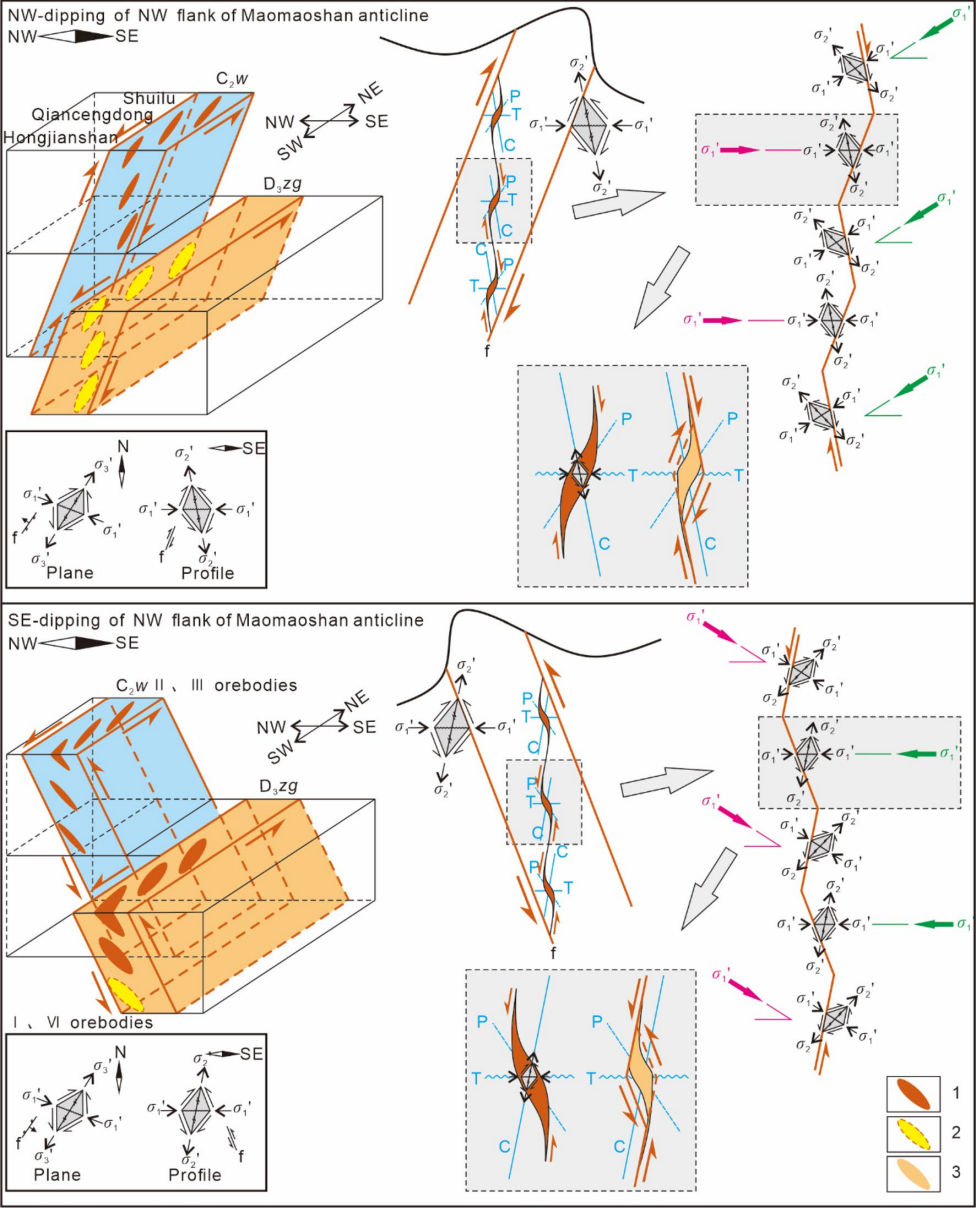
Schematic diagrams of the spatial distributions and the underlying mechanical model of the veins groups (orebodies) in the ore-bearing fault with different dips at the orebody groups scale. 1–Known orebody, 2–Predicted orebody, P is the long axis of the orebody centre, C is the compressional plane of the controlling orebody, and T is the tensile plane.

Since the series connection planes of orebodies indicate the planar shear direction along the ore-bearing fault, the long axis of each orebody is consistent with the sinistral torsional-compressional plane of the ore deposit. Therefore, the ore veins controlled by the NE-trending sinistral compressional-torsional ore-bearing fault zone in the ore deposit exhibit a right echelon planar distribution (Fig. 6).

#### Profile distribution patterns of the vein groups

The series connection plane of the profile on which the orebodies are obliquely distributed indicates the movement direction of the hanging wall and footwall of the compressional-torsional fault, and the ore veins are controlled by the opening space of the compressional-torsional ore-bearing fault zone; therefore, the ore veins controlled by the SE-dipping NE-trending sinistral compressional-torsional ore-bearing fault zone show a right echelon distribution in profile. The ore veins controlled by the NW-dipping NE-trending sinistral compressional-torsional ore-bearing fault zone exhibit a left echelon distribution in profile (Fig. 6).

Therefore, in northeastern Yunnan ore concentration area, the ore veins in the same ore-bearing fault zone show a left echelon distribution in plan view and a left or right echelon distribution in profile. The oblique distribution modes are directly controlled by the mechanical properties and dip of the ore-bearing fault.

### Orebody groups controlled by ore-forming faults at the deposit scale

**Figure 7:**
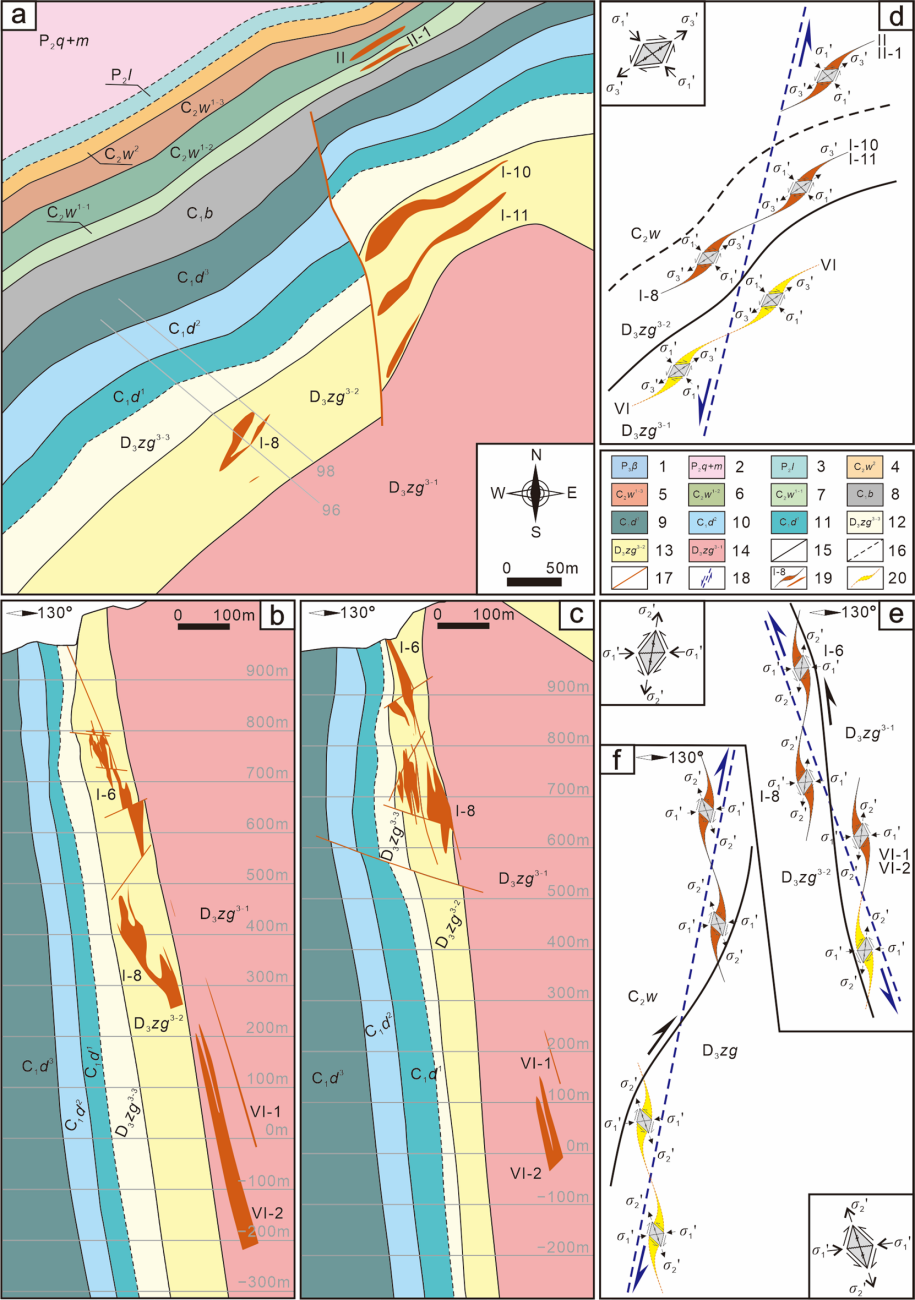
Schematic diagram of the spatial distributions and the underlying mechanical model of orebody groups controlled by ore-forming faults at the deposit scale. (**a**) Planar geological map of the 430 m middle segment of the Maoping Pb–Zn deposit, (**b**) Profile of the No. 96 exploration line of the Maoping Pb–Zn deposit, (**c**) Profile of the No. 98 exploration line of the Maoping Pb–Zn deposit, (**d**) Planar mechanics analysis diagram of the 430 m middle segment of the Maoping Pb–Zn deposit, (**e**) Mechanical analysis diagram of the profile of the Hedong region of the Maoping Pb–Zn deposit, and (**f**) Mechanical analysis diagram of the profile of the Hexi region of the Maoping Pb–Zn deposit. 1–Upper Permian basalt formation; 2–Middle Permian Qixia + Maokou Formation; 3–Lower Permian Liangshan Formation; 4–Middle Carboniferous Weining Formation; 5–Third submember of the first member of the middle Carboniferous Weining Formation; 6–Second submember of the first member of the middle Carboniferous Weining Formation; 7–First submember of the first member of the middle Carboniferous Weining Formation; 8–Lower Carboniferous Baizuo Formation; 9–Third member of the lower Carboniferous Datang Formation; 10–Second member of the lower Carboniferous Datang Formation; 11–First member of the lower Carboniferous Datang Formation; 12–Third submember of the third member of the Upper Devonian Zaige Formation; 13–second submember of the third member of the upper Devonian Zaige Formation; 14–First submember of the third member of the Upper Devonian Zaige Formation; 15–Conformal contact between strata; 16–Parallel non-conformal contact between strata; 17–Fault; 18–The series connection plane of orebody groups; 19–Known orebody; 20–Predicted orebody.

As discussed previously, Pb‒Zn deposits are often controlled by the main controlling faults in fault–fold structures (ore-conducting structures). Orebody groups controlled by ore-bearing faults in different ore-hosting strata form ore deposits or ore blocks, such as the I, II, III and VI orebody groups in the Hedong ore block of the Maoping Pb–Zn deposit and the Pb–Zn orebody groups in the Carboniferous Baizuo Formation and the Sinian Dengying Formation of the Pb–Zn deposits in the Huize mine. The analysis (Fig. 7) shows that in the Hedong ore block of the Maoping deposit, the series connection plane of the orebody groups in different strata controlled by the NE-trending compressional‒torsional fault is a torsional-compressional plane, which indicates the shear direction of the stress field where the ore deposit (orebody groups) is located; therefore, the orebody groups show a left echelon distribution in plan view. In profile, the Hexi ore block is located in the noninverted flank, the orebody groups are controlled by a compressional-torsional fissure zone, and their series connection plane is a torsional-compressional plane, which indicates the movement direction of the main fault. Thus, the orebody groups exhibit a right echelon distribution. The Hedong ore block is located in the inverted flank, and the orebody groups exhibit a left echelon distribution.

### Ore deposits controlled by ore field-scale structures

**Figure 8:**
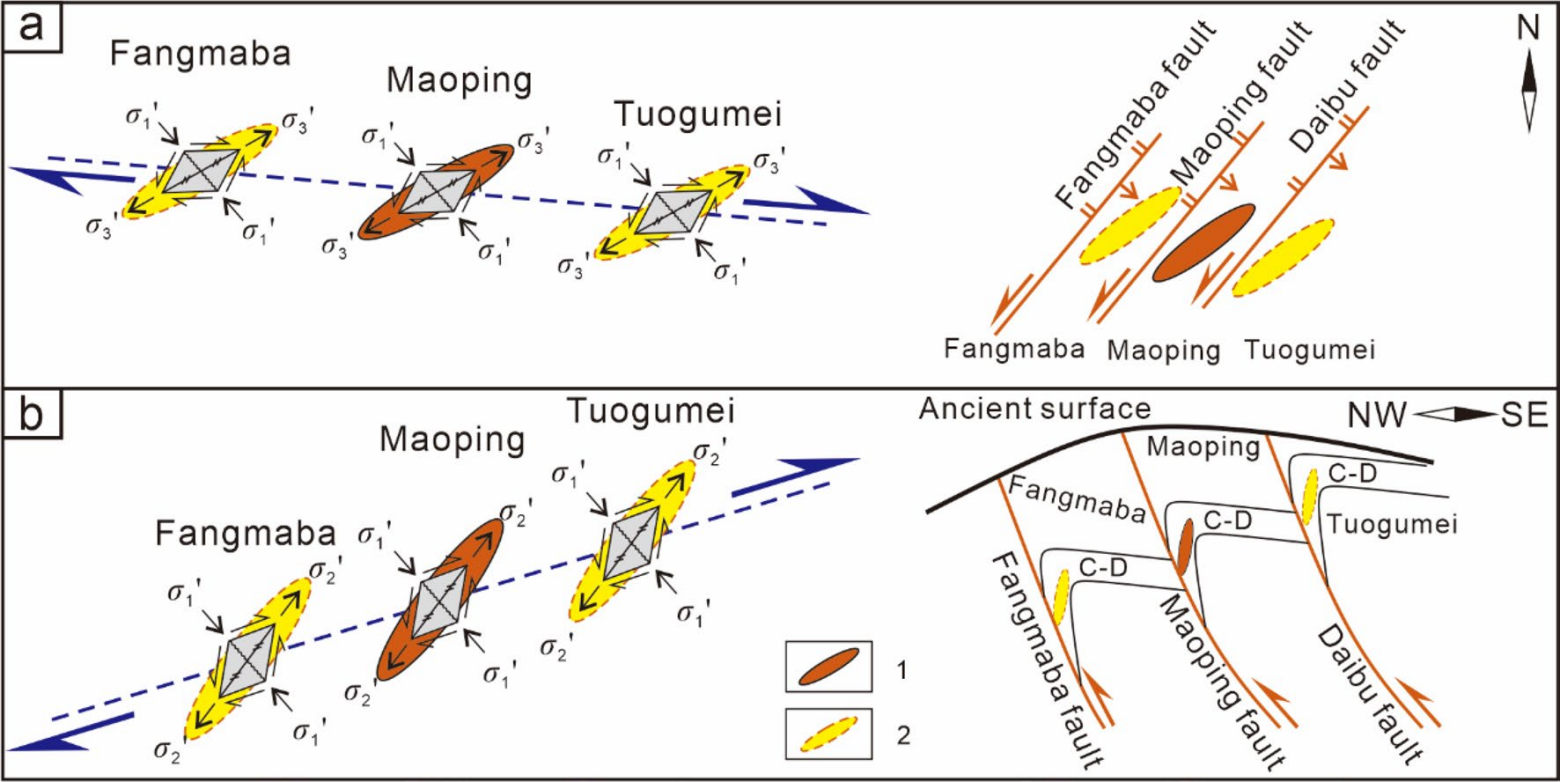
Schematic diagram of the spatial distribution and the underlying mechanical model of the deposits controlled by ore-forming faults at the ore field scale. (**a**) The oblique patterns of ore field scale of planes, (**b**) The oblique patterns of ore field scale of profile of a single orebody controlled by SE inclined interlayer fault, 1–Known orebody, 2–Predicted orebody.

For the Pb–Zn ore fields controlled by the NE-trending syncline fault–fold structure associations, such as the Maoping Pb–Zn ore field, which consists of deposits (ore occurrences) in the hanging walls of the Fangmaba Fault, Maoping Fault and Daibu Fault (Fig. 8), in plan view, the dextral lateral distribution pattern of each deposit is controlled by a dextral compressional‒torsional plane, and the series connection plane of ore deposits is a dextral torsional‒compressional plane, which indicates the principal compressive stress direction of the ore field. In profile, the lateral distribution pattern of the ore deposit is controlled by a compressional‒torsional plane, and the series connection plane of ore deposits is a torsional plane, which indicates that the movement direction of the major fault controlled the ore field. On the profile, the lateral distribution pattern of the deposits are controlled by a compression‒torsional plane, and the series connection plane of ore deposits is a torsional plane, which indicates that the movement direction of the major fault controlling the ore field, and the ore deposits exhibit a right echelon distribution.

### Oblique distribution patterns and the underlying mechanical model at different scales

In this study, the Maoping Pb–Zn deposit is taken as an example, and its oblique distribution patterns and the underlying mechanical model at different scales are summarized. The resulting conclusions can be directly applied to Pb–Zn deposits in the Ge-rich Pb‒Zn ore concentration area in northeastern Yunnan. Additionally, these conclusions can be extended to other nonmagmatic hydrothermal deposits worldwide, providing research ideas for the study of other magmatic hydrothermal deposits.

**Figure 9:**
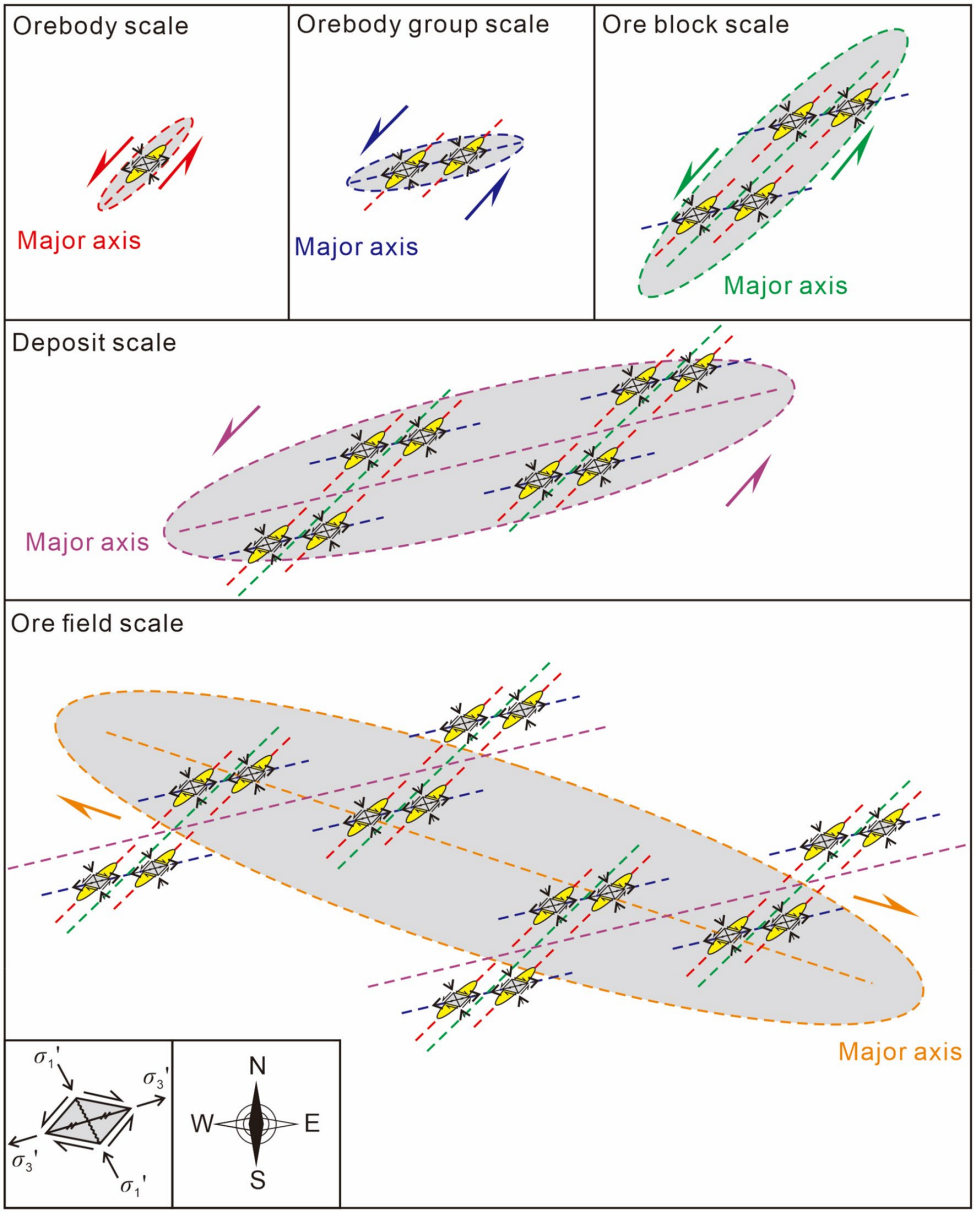
The oblique patterns and the underlying mechanical model at different scale in plan view.

**Figure 10:**
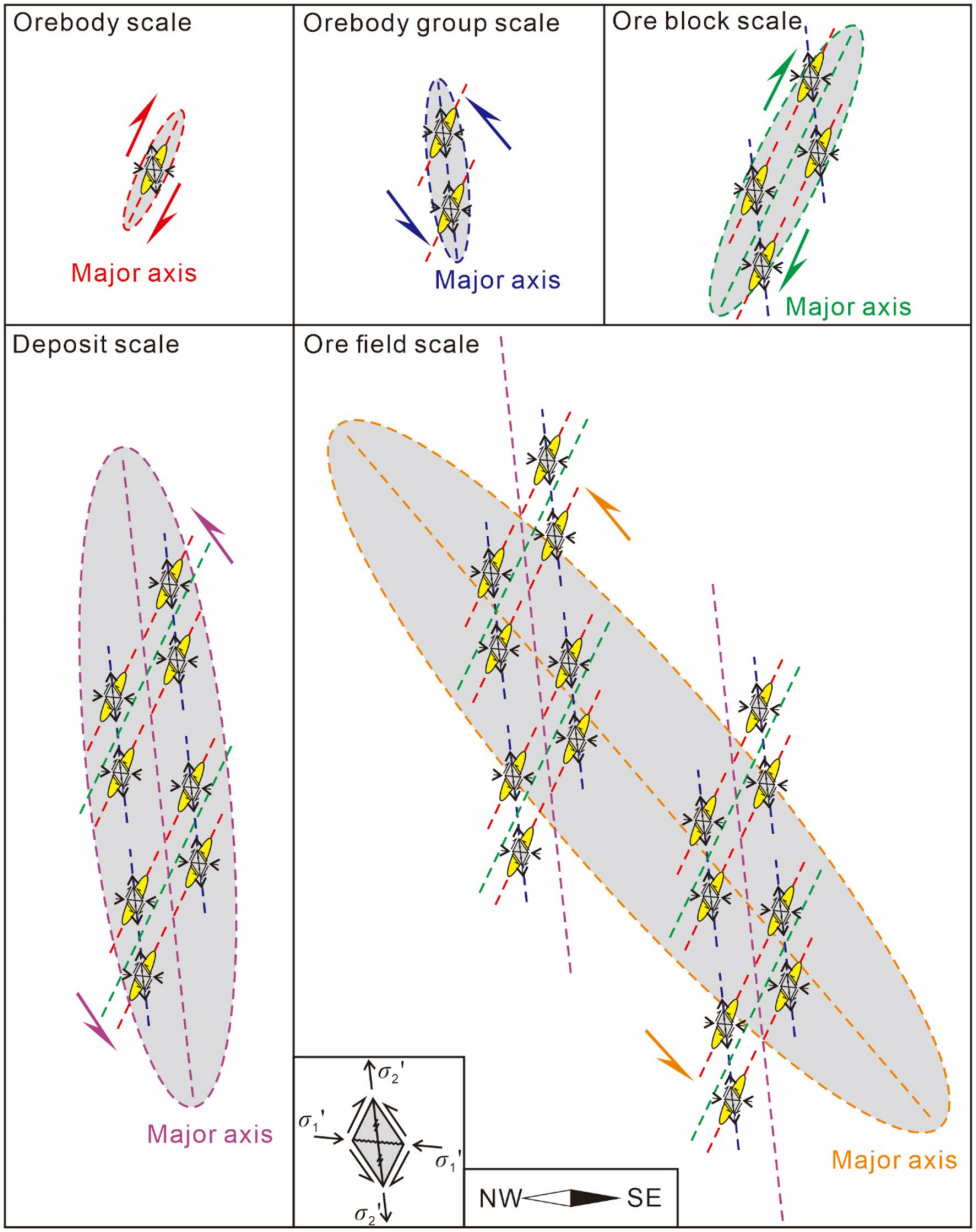
The oblique patterns and the underlying mechanical model at different scales in profile of a single orebody controlled by NW-dipping ore-forming fault.

**Figure 11:**
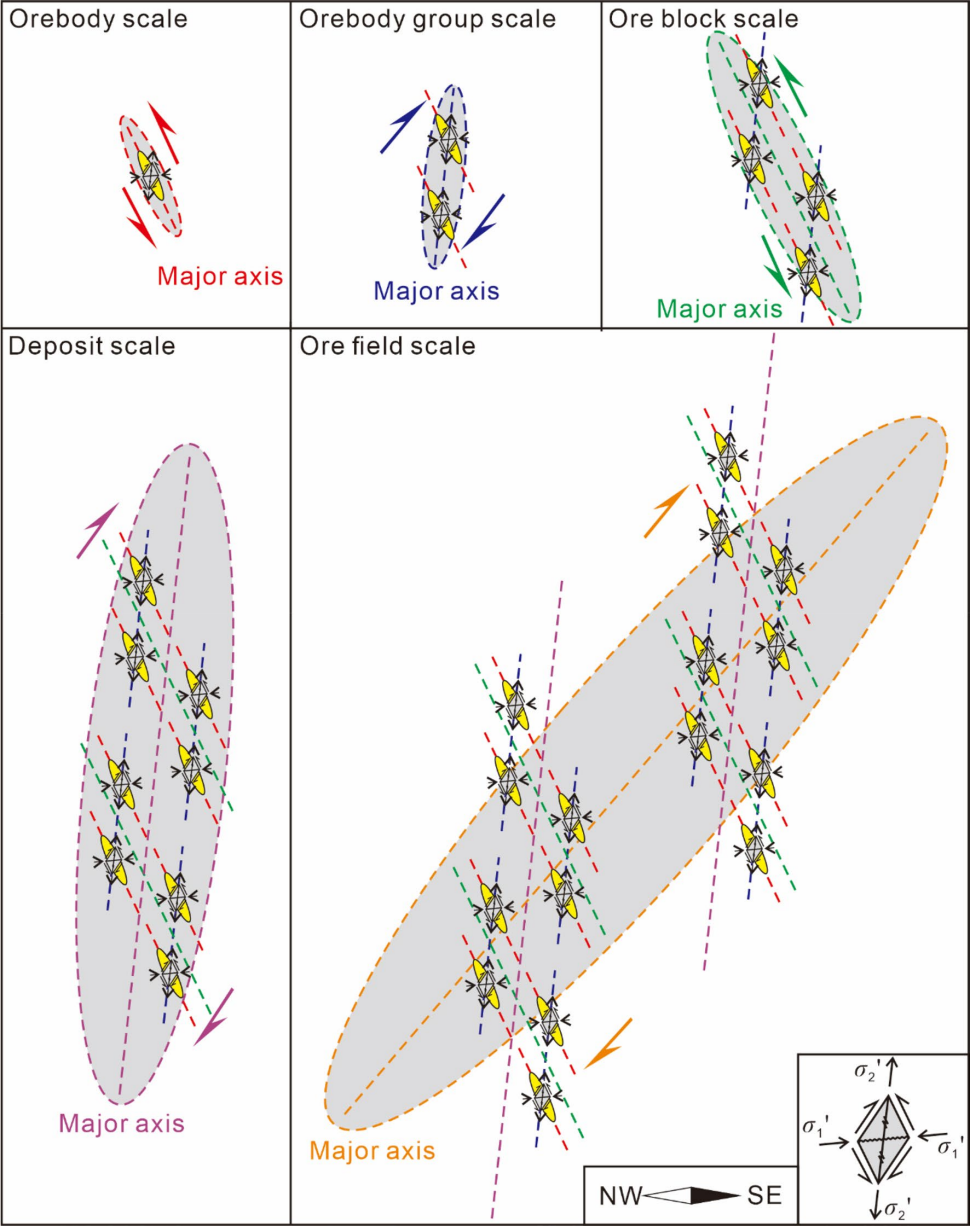
The oblique patterns and the underlying mechanical model at different scales in profile of a single orebody controlled by SE-dipping ore-forming fault.

An ore field consists of multiple deposits. A deposit consists of multiple ore blocks. An ore block consists of orebody groups with multiple ore-hosted formations. An orebody group consists of multiple orebodies (vein groups). The mechanical ore-controlling mechanisms vary at different scales. The mechanical ore-controlling mechanisms and oblique patterns of different scale planes and profiles are summarized in Tables 1, 2, and Figs. 9, 10, and 11.

**Table 1:** The underlying mechanical model of ore-controlling structures at different scale.

Plan			Profile			Contact
Scale	Object	Mechanical properties of ore-controlling	Scale	Object	Mechanical properties of ore-controlling	
Ore field	–	–	Ore field	Series plane	–	–
	Long axis	Dextral torsional-compressional plane		Long axis	Torsional-compressional plane	Long axis of ore field = series plane of deposit
Deposit	Series plane		Deposit	Series plane		
	Long axis	Dextral compressional-torsional plane		Long axis	Compressional-torsional plane	Long axis of deposit = series plane of ore block
Ore block	Series plane		Ore block	Series plane		
	Long axis	Sinistral torsional plane		Long axis	Torsional-compressional plane	Long axis of ore block = series plane of orebody group
Orebody group	Series plane		Orebody group	Series plane		
	Long axis	Sinistral compressional-torsional plane		Long axis	Compressional-torsional plane	Long axis of orebody group = series plane of orebody
Orebody	Series plane		Orebody	Series plane		
	Long axis	Sinistral torsional-compressional plane		Long axis	Torsional-compressional	–

**Table 2:** The oblique patterns at different scale.

Scale	Plan	Profile	
		NW-dipping interlayer fault	SE-dipping interlayer fault
Deposits of the field scale	Right echelon	Left echelon	Right echelon
Ore blocks of the deposit	Right echelon	Left echelon	Right echelon
Orebody groups of ore block	Left echelon	Right echelon	Left echelon
Orebodies of orebody groups	Right echelon	Left echelon	Right echelon

The Maoping Pb–Zn ore field is jointly controlled by three NE-trending compressional-torsional faults and NE-trending anticline structures, namely, the long axis of the ore field (the series plane of the deposits). In plan view, the ore field is controlled by the dextral torsional-compressional plane, and in profile, it is controlled by the torsional-compressional plane.

The Maoping deposit is controlled by a fault–fold structure composed of the NE-trending Maoping sinistral compressional-torsional fault and the NE-trending Maomaoshan compound inverted anticline, which is the long axis of the deposit (the series plane of the ore blocks). In plan view, the deposit is controlled by the dextral torsional-compressional plane, and in profile, it is controlled by the compressional-torsional plane.

The ore block is controlled by a N–S-trending sinistral torsional fault and a NNW-trending sinistral torsional fault, which form the long axis of the ore block (the series plane of the orebody groups). In plan view, it is controlled by the sinistral torsional-compressional plane, and in profile, it is controlled by the torsional-compressional plane.

The orebody group is controlled by the NE-trending interlayer compressional-torsional fault within the lithological combination of clastic rock cover and carbonate rock, which is the long axis of the orebody group (the series plane of the orebodies). In plan view, the orebody group is controlled by the sinistral compressional-torsional plane, and in profile, it is controlled by the compressional-torsional plane.

The orebody is controlled by the opening space of the main pressure plane within the NE-trending interlayer compressional-torsional fault, which is the long axis of the orebody group (the series plane of the orebodies). In plan view, the orebody is controlled by the dextral torsional-compressional plane, and in profile, it is controlled by the torsional-compressional plane.

The mechanical ore-controlling mechanisms at different scales under the same principal compressive stress within the scope of the ore field led to the formation of different oblique patterns in plan and profile views.

The orebodies at the scale of the orebody group are in a left echelon arrangement in plan view. The NW-dipping NE-trending fault is a sinistral compressional-torsional ore-bearing fault, with the orebodies exhibiting a left echelon arrangement. The SE-dipping NE-trending fault is a sinistral compressional-torsional ore-bearing fault, with the orebodies exhibiting a right echelon arrangement.

The orebody groups at the scale of the ore block exhibit a left echelon arrangement in plan view. The NW-dipping NE-trending fault is a sinistral compressional-torsional ore-bearing fault, with the orebodies exhibiting a right echelon arrangement. The SE-dipping NE-trending fault is a sinistral compressional-torsional ore-bearing fault, with the orebodies exhibiting a left echelon arrangement.

The ore blocks at the scale of the deposit exhibit a right echelon arrangement in plan view. The NW-dipping NE-trending fault is a sinistral compressional-torsional ore-bearing fault, with the orebodies exhibiting a left echelon arrangement. The SE-dipping NE-trending fault is a sinistral compressional-torsional ore-bearing fault, with the orebodies exhibiting a right echelon arrangement.

The deposits at the scale of the ore field exhibit a right echelon arrangement in plan view. The NW-dipping NE-trending fault is a sinistral compressional-torsional ore-bearing fault, with the orebodies exhibiting a left echelon arrangement. The SE-dipping NE-trending fault is a sinistral compressional-torsional ore-bearing fault, with the orebodies exhibiting a right echelon arrangement.

### Deep exploration and targeting of ore deposits and discovery of concealed orebody groups

#### Prediction of orebody group distribution at the deposit scale

**Figure 12:**
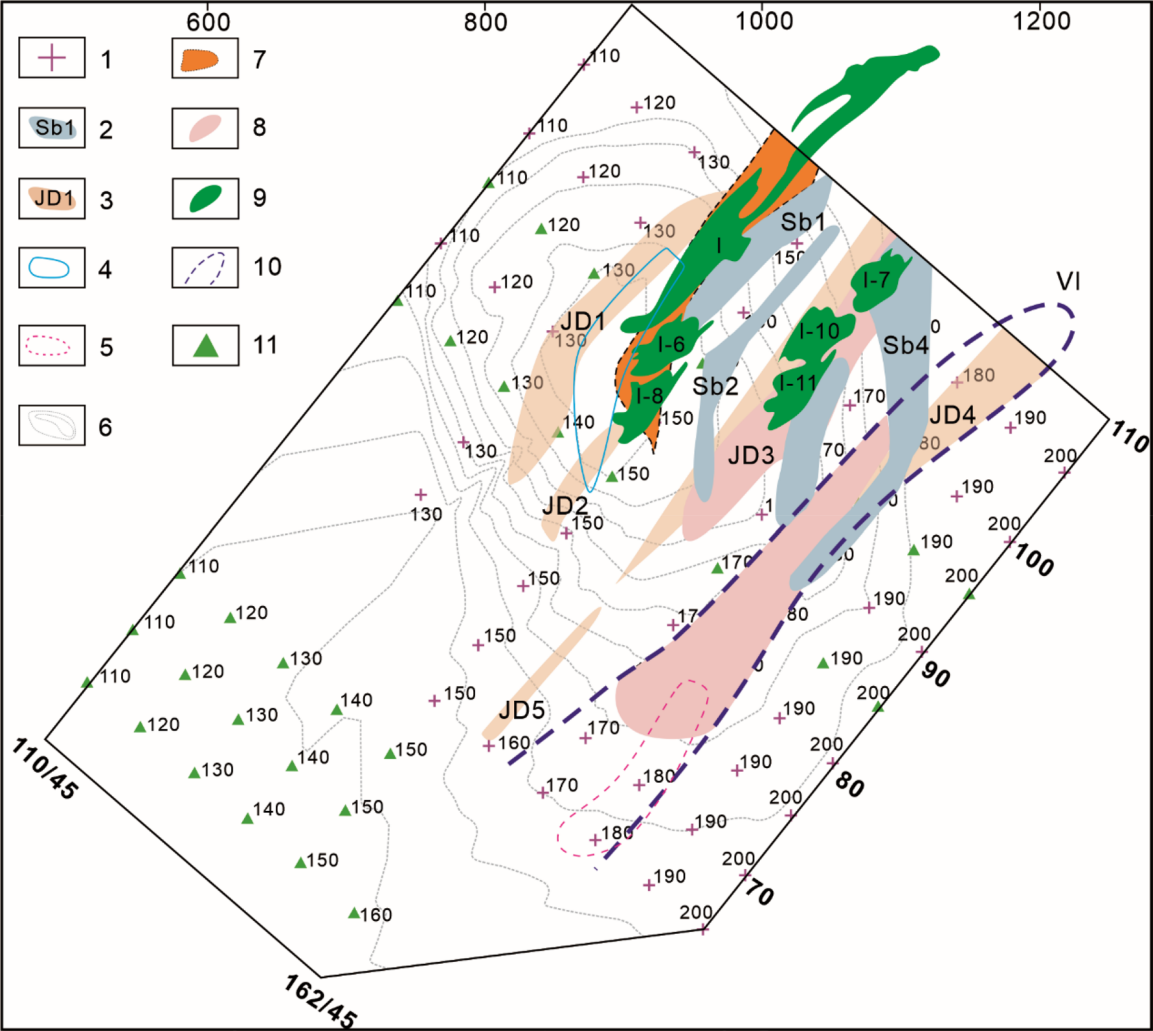
Comprehensive TEM and IP geophysical prospecting anomalies and planar prediction diagram of the mineralized zones in the Maoping Pb–Zn deposit. 1–IP measuring point; 2–TEM low resistance anomaly; 3–IP high susceptibility anomaly; 4–Inferred orebody horizontal projection from charging point; 5–Apparent charging rate of IP profile; 6–Anomaly at the charging point; 7–Speculated extension position of No. I orebody; 8–Key prospecting target area; 9–Projection of newly discovered orebody; 10–Predicted mineralized zone; 11–Charging measuring point. Comprehensive TEM and IP geophysical prospecting anomalies and planar prediction diagram of the mineralized zones in the Maoping Pb–Zn deposit. © 2024 by Runsheng Han is licensed under Attribution 4.0 International.

**Figure 13:**
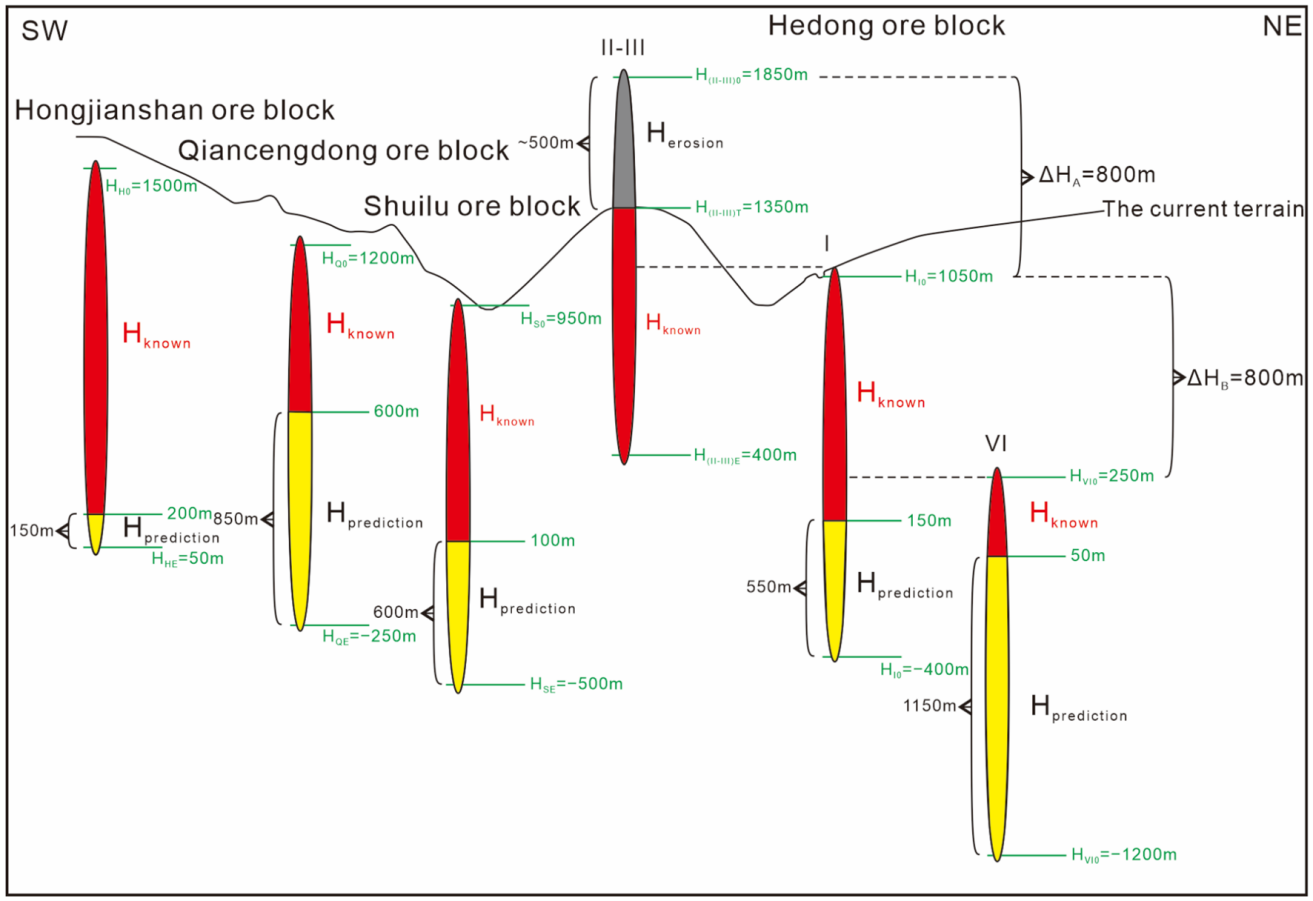
Schematic diagram of the semiquantitatively predicted extension depths of the orebody groups in each mineralized zone of the Maoping Pb–Zn deposit. Red–Known orebodies, Yellow–Predicting orebodies, Grey–Erosion orebodies.

During ore prospecting in the deep part of the Maoping Pb–Zn deposit in Zhaotong, based on the left echelon distribution pattern of the orebody groups on the plane, orebody groups with a left echelon distribution are predicted to be present on the SE sides of the I, I-1, I-6, and I-8 orebody groups and the I-7, I-10, and I-11 orebody groups of the Hedong ore block (Figs. 6, 7). This prediction is consistent with the inferred geophysical anomaly zone (Fig. 12). Engineering verification revealed the presence of the VI orebody group. In addition, based on the vertical oblique distribution pattern of the four mineralized zones in the Hedong ore block and the three mineralized zones in the Hexi ore blocks, the depth characteristics of the orebody groups and the locations of occurrence of new orebody groups in each mineralized zone can be semiquantitatively predicted (Fig. 13).

Based on the tectonic ore-controlling pattern, positioning characteristics and theoretical assumptions of the orebody groups, the erosion depths of the orebody groups can be inferred.

1. Based on the hierarchical ore-controlling pattern of the ore deposit structure, the main orebody groups controlled by the Maoping fault–fold structure mainly occur in the hanging wall of the Maoping fault, and the vertical depth is far greater than the along-strike length, which indicates that the extension depth of the main fault H_f_ is equivalent to the extension depth of the main orebody groups H_o_:
  1 $${\text{H}}_{{\text{f}}} = {\text{H}}_{{\text{o}}} .$$
2. The equidistant distribution patterns of the ore-hosting structure and the distance occurrence pattern of the main orebody group at the same depth are consistent with each other, and the vertical extension distances of the main orebody groups are equivalent.
  Vertical extension of the main orebody groups
  2 $${\text{H}}_{{\text{I}}} = {\text{H}}_{{{\text{II}} - {\text{III}}}} = {\text{H}}_{{{\text{VI}}}} = {\text{H}}_{{\text{H}}} = {\text{H}}_{{\text{Q}}} = {\text{H}}_{{\text{S}}} .$$
  Distance between the main orebody groups at the same depth:
  3 $$\Delta {\text{H}}_{{\text{A}}} = \Delta {\text{H}}_{{\text{B}}} .$$
3. The surface exposure elevation H_I0_ of the No. I orebody in the No. I mineralized zone is approximately 1050 m; the exposure elevation H_VI0_ of the No. VI orebody group is approximately 250 m; the exposure elevation H_H0_ of the Hongjianshan ore block is approximately 250 m; the exposure elevation H_Q0_ of the initial segment of the Qiancengdong ore block is approximately 1200 m; the exposure elevation H_S0_ of the initial segment of the Shuilu Ore block is approximately 950 m; and the maximum surface exposure elevation of the II and III orebody groups is approximately
  4 $${\text{H}}_{{\left( {{\text{II}} - {\text{III}}} \right){\text{T}}}} = {135}0{\text{ m}}.$$
4. The erosion depths of the surface in the same area are comparable.
  Therefore, in combination with (3), the relative vertical difference in the occurrence depths of the main orebodies is
  5 $$\Delta {\text{H}}_{{\text{A}}} = \Delta {\text{H}}_{{\text{B}}} = {\text{H}}_{{{\text{I}}0}} {-}{\text{H}}_{{{\text{VI}}0}} = {1}0{5}0 - {25}0 = {8}00{\text{ m}}.$$
  Vertical erosion depth of the orebody
  6 $${\text{H}}_{{{\text{erosion}}}} \ge \Delta {\text{H}}_{{\text{A}}} - \left( {{\text{H}}_{{\left( {{\text{II}} - {\text{III}}} \right){\text{T}}}} {-}{\text{H}}_{{{\text{I}}0}} } \right) = {8}00 - \left( {{135}0 - {1}0{5}0} \right) = {5}00{\text{ m}}.$$

Based on the above analyses, according to the characteristics of the tectonic ore-controlling pattern and orebody group erosion depth, the pinch-out elevation of the maximum extension depth of the orebody group and the maximum depth of the re-extension of the main mineralized belts in the mining area are proposed. This prediction is an overall inference based on the ore-controlling structure pattern and orebody position pattern, and the vertical error is 50–100 m.

1. Based on the original exposure elevations of the II and III orebody groups (when the ore deposit was formed),
  7 $${\text{H}}_{{\left( {{\text{II}} - {\text{III}}} \right)0}} = {135}0 - {5}00 = {185}0{\text{ m}}.$$
  The exposure elevation H_(II–III)E_ of the deep pinch-out end is 400–500 m (calculated using 400 m); therefore, it can be inferred that the elevation difference in the initial extension depth of the II and III orebody groups can be calculated as follows:
  8 $${\text{H}}_{{{\text{II}} - {\text{III}}}} = {\text{H}}_{{\left( {{\text{II}} - {\text{III}}} \right)0}} - {\text{H}}_{{\left( {{\text{II}} - {\text{III}}} \right){\text{E}}}} = {185}0 - {4}00 = {145}0{\text{ m}}.$$
  Therefore, in combination with (2), the vertical extension of the main orebody group is
  9 $${\text{H}}_{{\text{I}}} = {\text{H}}_{{{\text{VI}}}} = {\text{H}}_{{\text{H}}} = {\text{H}}_{{\text{Q}}} = {\text{H}}_{{\text{S}}} = {\text{H}}_{{{\text{II}} - {\text{III}}}} = {145}0{\text{ m}}.$$
2. Pinch-out elevation of the maximum depth extension of the No. I orebody group:
  10 $${\text{H}}_{{{\text{IE}}}} = {\text{H}}_{{{\text{I}}0}} {-}{\text{H}}_{{\text{I}}} = {1}0{5}0 - {145}0 = - {4}00{\text{ m}}.$$
  The currently controlled elevation difference in prospecting projects is approximately 850–950 m, and the minimum controlled elevation under current engineering control is approximately 100–200 m. Accordingly, the maximum depth of the re-extension of this mineralized zone at the current occurrence elevation is 500–600 m.
  11 $${\text{Minimum controlled elevation }}\left( {{1}00 - {2}00{\text{ m}}} \right) - {\text{pinch}} - {\text{out elevation H}}_{{{\text{IE}}}} \left( { - {4}00{\text{ m}}} \right) = {5}00 - {6}00{\text{ m}}.$$
3. Pinch-out elevation of the No. VI orebody group:
  12 $${\text{H}}_{{{\text{VIE}}}} = {\text{H}}_{{{\text{VI}}0}} - {\text{H}}_{{{\text{VI}}}} = {25}0 - {145}0 = - {12}00{\text{ m}}.$$
  At present, the controlled elevation difference is approximately 150–250 m, and the minimum controlled elevation is 0–100 m. Therefore, the calculated maximum depth of the re-extension of the mineralized zone where this orebody group is located at the current elevation is 1200–1300 m.
  13 $${\text{Minimum controlled elevation }}\left( {0 - {1}00{\text{ m}}} \right) - {\text{pinch}} - {\text{out elevation H}}_{{{\text{VIE}}}} \left( { - {12}00{\text{ m}}} \right) = {12}00 - {13}00{\text{ m}}.$$
4. Pinch-out elevation of the orebody group in the Hongjianshan ore block in Hexi:
  14 $${\text{H}}_{{{\text{HE}}}} = {\text{H}}_{{{\text{H}}0}} - {\text{H}}_{{\text{H}}} = {15}00 - {145}0 = {5}0{\text{ m}}.$$
  Currently, the elevation difference in this orebody group is controlled at approximately 1250–1300 m, and the minimum controlled elevation is 200–250 m. Therefore, the calculated maximum depth of the mineralized zone where this orebody group is located at the current occurrence elevation is 150–200 m:
  15 $${\text{Minimum controlled elevation }}\left( {{2}00 - {25}0{\text{ m}}} \right) - {\text{pinch}} - {\text{out elevation}}\;{\text{H}}_{{{\text{HE}}}} \left( {{5}0{\text{ m}}} \right) = {15}0 - {2}00{\text{ m}}.$$
5. The pinch-out elevation of the orebody group at the maximum depth extension in the Qiancengdong ore block:
  16 $${\text{H}}_{{{\text{QE}}}} = {\text{H}}_{{{\text{Q}}0}} - {\text{H}}_{{\text{Q}}} = {12}00 - {145}0 = - {25}0{\text{ m}}.$$
  The controlled elevation difference of this group of orebodies is approximately 600 m, and the minimum controlled elevation is approximately 600 m. Therefore, it is inferred that the maximum re-extension depth of the mineralized zone where this orebody group is located at the current elevation is approximately 850 m.
  17 $${\text{Minimum controlled elevation }}\left( {{6}00{\text{ m}}} \right) - {\text{pinch}} - {\text{out elevation}}\;{\text{H}}_{{{\text{QE}}}} \left( { - {25}0{\text{ m}}} \right) = {85}0{\text{ m}}.$$
6. Pinch-out elevation of the orebody group in the Shuilu ore block at the maximum depth extension:
  18 $${\text{H}}_{{{\text{SE}}}} = {\text{H}}_{{{\text{S}}0}} - {\text{H}} = {95}0 - {145}0 = - {5}00{\text{ m}}.$$
  The controlled elevation difference of this orebody group is approximately 800–850 m, with a minimum controlled elevation of approximately 100 m. Therefore, it is inferred that the maximum re-extension depth of the mineralized zone where this orebody group is located at the current elevation is 600–650 m.
  19 $${\text{Minimum controlled elevation }}\left( {{1}00{\text{ m}}} \right) - {\text{pinch}} - {\text{out elevation H}}_{{{\text{SE}}}} \left( { - {5}00{\text{ m}}} \right) = {6}00{\text{ m}}.$$

#### Prediction of the spatial distribution of orebodies based on the lateral distribution of orebody groups

As mentioned above, among the four ore blocks of the Maoping Pb–Zn deposit, the Nos. I and VI orebody groups in the Hedong ore block and the Qiancengdong and Shuilu ore blocks in the Hexi region have great deep resource potential. The No. VI orebody group has a large extension depth and greater resource potential. Based on the oblique distribution patterns of the ore vein group in plan view and in profile, the occurrence locations of the new ore veins were predicted (Figs. 6, 7). In the Nos. I and VI orebody groups in the Hedong ore block, the key focus should be the predicted orebodies showing a right echelon distribution relative to the deep parts of the known orebodies (such as I-8, I-11, VI-1, VI-2, etc.). Similarly, in the Qiancengdong and Shuilu ore blocks in the Hexi ore blocks, the key focus should be the predicted orebodies showing a left echelon distribution relative to the known orebodies. In addition, attention should be given to predicting the locations of the sinistral lateral orebodies in plan view with ores hosted in D_3_zg in the Hongjianshan, the Qiancengdong and the Shuilu ore blocks in Hexi (Figs. 6, 7).

## Conclusions

1. Through the systematic analysis of the oblique distribution patterns of orebodies (orebody groups) controlled by structures at different scales and by the underlying mechanical model, the oblique distributions of ore veins, orebody groups and ore deposits in three-dimensional space are directly controlled by the mechanical properties, kinematic characteristics and tectonic stress field of ore-controlling/ore-forming fault structures of different sequences of the mineralization period.
2. Based on the oblique distribution patterns of orebody groups in the ore deposits, the depth characteristics of the orebody groups in each mineralized zone and the occurrence locations of new orebody groups can be predicted semiquantitatively.
3. Based on the oblique distribution patterns of ore deposits, orebody groups and orebodies and the underlying mechanical model, the peripheral and deep parts of the Maoping Pb–Zn ore field, ore deposits, and orebody groups in northeastern Yunnan ore concentration area were successfully predicted. Research has shown that the oblique distribution patterns of orebody groups controlled by structures of different scales can be widely applied to nonmagmatic hydrothermal deposits and that they can provide information on magmatic hydrothermal deposits controlled by structures.

## Data Availability

All data generated or analysed during this study are included in this published article.

## References

[1] Han RS, Wu P, Wang F, Zhou GM, Li WY, Qiu WL (2019). “Four Steps Type" ore–prospecting method for deeply concealed hydrothermal ore deposits—A case study of the Maoping Zn–Pb (Ag–Ge) deposit in southwestern China. Geotect. Metal.

[2] Liu HC, Lin WD (1999). A Study on the Regularity of Pb–Zn Silver Deposits in Northeast Yunnan.

[3] Han RS, Hu YZ, Wang XK, Hou BH, Huang ZL, Chen J, Wang F, Wu P, Li B, Wang HJ, Dong Y, Lei L (2012). Mineralization model of rich Ge–Ag–bearing Zn–Pb polymetallic deposit concentrated district in Northeastern Yunnan, China. Acta Geol. Sinica.

[4] Leach DL, Bradley DC, Huston D, Pisarevsky SA, Taylor RD, Gardoll SJ (2010). Sediment-hosted Pb–Zn deposits in earth history. Econ. Geol..

[5] Zhao D, Han RS, Wang L, Ren T, Wang JS, Zhang XP, Ding JJ (2021). Genesis of the Lehong large zinc–lead deposit in northeastern Yunnan, China: Evidences from geological characteristics and C-H–O–S–Pb isotopic compositions. Ore Geol. Rev..

[6] Han RS, Liu CQ, Huang ZL, Chen J, Ma DY, Li Y (2001). Genesis modeling of Huize Pb–Zn ore deposit in Yunnan. Acta Mineralogica Sinica.

[7] Han RS, Wu P, Zhang Y, Huang ZL, Wang F, Zhou GM, Shi ZL, Zhang CQ (2022). New progresses of metallogenic theory for rich Zn–Pb–(Ag–Ge) deposits in the Sichuan–Yunnan–Guizhou Triangle (SYGT) area, Southwestern Tethys. Acta Geologica Sinica.

[8] Miao Y, Li WC, Zhou JX, Luo K, Zhou Y, Chen SM, Fan ZY, Pan JR (2023). Geology, geochemistry and genesis of the giant Maoping carbonate-hosted Pb–Zn–(Ag–Ge) deposit in northeastern Yunnan Province, SW China. Ore Geol. Rev..

[9] Zhang LS (1998). Several geological problems of lead zinc deposits dominated by carbonate rocks on the Eastern Margin of the Kangdian Axis. Deposits.

[10] Huang ZL, Chen J, Han RS, Li WB, Liu CQ, Zhang ZL, Yang HL (2004). Geochemistry and Ore-Formation of the Huize Giant Pb–Zn Deposit, Yunnan, Province, China: discussion on the Relationship Between Emeishan Flood Basalts and Pb–Zn Mineralization.

[11] Zhang CQ, Mao JW, Liu F, Li HM (2005). K-Ar dating of altered clay minerals from Huize Pb–Zn deposit in Yunnan Province and its geological significance. Deposits.

[12] Mao JW, Li XF, Li HM, Qu XM, Zhang CQ, Xue CJ, Wang ZL, Yu JJ, Zhang ZH, Feng CY, Wang RT (2005). Types and characteristics of endogenetic metallic deposits in orogenic belts in China and their metallogenic processes. Acta Geologica Sinica.

[13] Wang DH, Chen ZH, Chen YC, Tang JX, Li JK, Ying LJ, Wang CH, Liu SB, Li LX, Qin Y, Li HQ, Qu WJ, Wang YB, Chen W, Zhang Y (2010). New data of the rock-forming and ore-forming chronology for China’s important mineral resources areas. Acta Geologica Sinica.

[14] Han RS, Chen J, Huang ZL, Ma DY, Xue CD, Li Y (2006). Dynamics of tectonic ore-forming process and localization-prognosis of concealed orebodies—As exemplified by the Huize Surper-large Zn–Pb–(Ag–Ge) District.

[15] Han RS, Wang F, Hu YZ, Wang XK, Ren T, Qiu WL, Zhong KH (2014). Metallogenic tectonic dynamics and chronology constrains on the Huize-type (HZT) germanium-rich silver-zinc-lead deposits. Geotectonica et Metallogenia.

[16] Tu GZ (1984). Geochemistry of Stratified Deposits in China.

[17] Chen SJ (1986). Research on the genesis of Pb–Zn ore-deposits in western Guizhou and northeastern Yunnan. Geol. Guizhou.

[18] Zhang Y, Han RS, Ding X, Wang YR, Wei PT (2019). Experimental study on fluid migration mechanism related to Pb–Zn super–enrichment: Implications to mineralization mechanisms of the Pb–Zn deposits in the Sichuan–Yunnan–Guizhou, SW China. Ore. Geol. Rev..

[19] Han RS, Liu CQ, Huang ZL, Chen J, Ma DY, Lei L, Ma GS (2007). Geological features and origin of the Huize carbonate-hosted Zn–Pb–(Ag) district, Yunnan, South China. Ore Geol. Rev..

[20] Lv YH, Han RS, Ren T, Qiu WL, Rang H, Gao Y (2015). Ore-controlling characteristics of fault structures and their relations to mineralization at Fulechang Zn–Pb mining district in deposit concentration district of northeastern Yunnan, China. Geoscience.

[21] Huang KN, Opdyke ND (2015). Post-folding magnetization of the Triassic rocks from western Guizhou and southern Yunnan provinces: New evidence for large clockwise rotations in the Simao Terrane. Earth Planet. Sci. Lett..

[22] Li ZT, Han RS, Yan QW (2017). Mineralization-alteration zoning regularity and structural ore-controlling role in the Huize super-large sized Ge–Ag-rich Pb–Zn deposit, Yunnan Province. Geol. China.

[23] Ni CZ, Zhang ST, Chen Z, Yan YF, Li YJ (2017). Mapping the spatial distribution and characteristics of lineaments using fractal and multifractal models: A case study from Northeastern Yunnan Province, China. Sci. Rep..

[24] Cui JH, Han RS, Wang JS, Ren T, Wu YT, Zhao D (2018). Generation and development of structures and their controls on ore mineralization in the Lehong zinc–lead deposit, Northeastern Yunnan. Geotectonica et Metallogenia.

[25] Wu JB, Han RS, Zhou GM, Shi ZL, Zhang Y, Sun BT, Zhong H, Liu XK, Zuo JG (2023). Structural ore-controlling and deep prospecting direction of the Maoping Pb–Zn deposit in Northeastern Yunnan, China. Geotectonica et Metallogenia.

[26] Han RS, Chen J, Gao DR, Huang ZL, Ma DY, Li Y, Zhao DS (2003). Application of tectono-geochemical ore-finding method in orientation prognosis of concealed ores. Geol. Prospecting.

[27] Han RS, Chen J, Wang F, Wang XK, Li Y (2015). Analysis of metal–element association halos within fault zones for the exploration of concealed orebodies—A case study of the Qilinchang Zn–Pb–(Ag–Ge) deposit in the Huize mine district, northeastern Yunnan, China. J. Geochem. Exploration.

[28] Qiu NS, Liu S (2018). Uplift and denudation in the continental area of China linked to climatic effects: Evidence from apatite and zircon fission track data. Sci. Rep..

[29] Schellart WP, Chen Z, Strak V, Duarte JC, Rosas FM (2019). Pacific subduction control on Asian continental deformation including Tibetan extension and eastward extrusion tectonics. Nat. Commun..

[30] Bolotov IN, Pasupuleti R, Subba Rao NV, Unnikrishnan SK, Chan N, Lunn Z, Win T, Gofarov MY, Kondakov AV, Konopleva ES, Lyubas AA, Tomilova AA, Vikhrev IV, Pfenninger M, Düwel SS, Feldmeyer B, Nesemann HF, Nagel KO (2022). Oriental freshwater mussels arose in East Gondwana and arrived to Asia on the Indian Plate and Burma Terrane. Sci. Rep..

[31] Tian EY, Xiao B, Zhang JJ, Qin JH, Lai Y, Ke K, Tian KZ, Zhang JW, Qin Y, Chen YL, Gong DX (2023). Origin and metal-enrichment mechanism of sedimentary rare earth element deposits in Yunnan and Guizhou provinces, western Yangtze Block, China. Ore Geol. Rev..

[32] Wang LF, Barbot S (2023). Three-dimensional kinematics of the India-Eurasia collision. Commun. Earth Environ..

[33] Shang JX, Feng MY, Wang XZ, Zhang BJ, Xu L, Liu XH (2023). Alteration effects of karstification and hydrothermalism on middle Permian Qixia formation at the Wulong section, South China. Sci. Rep..

[34] Flament N, Williams S, Müller RD, Gurnis M, Bower DJ (2017). Correspondence: Reply to ‘Numerical modelling of the PERM anomaly and the Emeishan large igneous province’. Nat. Commun..

[35] Wu JB, Pi QH, Zhu B, Hu YH, Li G, Wei CW (2020). Late Cretaceous-Cenozoic exhumation of Northwestern Guangxi (China) and tectonic implications: Evidence from apatite fission track dating. Geochemistry.

[36] Sun JC, Han RS (2016). Theory and Method of Minefield Geomechanics.

